# Chronic pain and its relationship with work participation

**DOI:** 10.1093/occmed/kqag079

**Published:** 2026-09-07

**Authors:** H Blake, W J Chaplin, V Abbott-Fleming, G Taylor, P McNamee, D A Walsh, K Walker-Bone, D F McWilliams

**Affiliations:** School of Health Sciences, University of Nottingham, Queen's Medical Centre, Nottingham, NG7 2HA, UK; Arthritis UK Pain Centre, University of Nottingham, Nottingham, Clinical Sciences Building, City Hospital, NG5 1PB, UK; NIHR Nottingham Biomedical Research Centre, Queens Medical Centre, Nottingham, NG7 2UH, UK; School of Health Sciences, University of Nottingham, Queen's Medical Centre, Nottingham, NG7 2HA, UK; NIHR Nottingham Biomedical Research Centre, Queens Medical Centre, Nottingham, NG7 2UH, UK; British Pain Society, London, EC1V 2NX, UK; College of Medicine and Health, University of Exeter, Exeter, EX1 2LU, UK; Health Economics Research Unit, Institute of Applied Health Sciences, University of Aberdeen, Aberdeen, AB25 2ZD, UK; Arthritis UK Pain Centre, University of Nottingham, Nottingham, Clinical Sciences Building, City Hospital, NG5 1PB, UK; NIHR Nottingham Biomedical Research Centre, Queens Medical Centre, Nottingham, NG7 2UH, UK; School of Medicine, University of Nottingham, Nottingham, NG7 2UH, UK; Monash Centre for Occupational and Environmental Health, Monash University, Clayton, Victoria 3800, Australia; Arthritis UK Pain Centre, University of Nottingham, Nottingham, Clinical Sciences Building, City Hospital, NG5 1PB, UK; NIHR Nottingham Biomedical Research Centre, Queens Medical Centre, Nottingham, NG7 2UH, UK; School of Medicine, University of Nottingham, Nottingham, NG7 2UH, UK

## Abstract

**Background:**

Chronic pain affects around 28 million people in the United Kingdom and is a major public health challenge with a substantial economic and social burden. Employers lack awareness of the impacts of chronic pain on work and employee wellbeing which hinders provision of workplace support.

**Aims:**

To examine the relationship between chronic pain and work participation measured in working age adults in England.

**Methods:**

Analysis of online survey data (*n* = 310) from a cluster-randomized feasibility trial (Pain-at-Work), including measures of pain severity, participant-reported work participation measures, psychological wellbeing and work-related social support. Multivariable regression analyses examined the relationship between pain severity, work-related and psychological measures, adjusted for age, gender and Body Mass Index classification.

**Results:**

On average, 2.2 h/week (SD = 6.4) were lost due to health-related issues. Percentage of work productivity loss was 46.4% (SD = 27.0), mean health-related absenteeism was 7.6% (SD = 20.4), while presenteeism averaged 44.3% (SD = 26.3). Increased pain severity was associated with increased presenteeism (*β* = 3.67, 95% CI 2.08–5.27, *P *< 0.001), higher work productivity loss (*β* = 4.79, 95% CI 2.13–7.44, *P = *0.001), increased activity impairment (*β* = 4.93, 95% CI 3.41–6.44, *P *< 0.001) and increased work limitations in the physical demand domain (*β* = 3.92, 95% CI 2.64–5.21, *P *< 0.001).

**Conclusions:**

Chronic pain adversely impacts multiple aspects of work participation and psychological wellbeing, with effects worsening as pain severity increases. Employers can help mitigate this through tailored support and job design since hybrid working may be protective in contexts where it is viable.

## Teaser

Many employers lack awareness of the impact of chronic pain on work and wellbeing, which can be a barrier to employees with chronic pain accessing support. This study shows that increasing pain severity is associated with psychological ill-health, lower work ability, greater work presenteeism and work productivity loss in working-age adults with chronic pain from diverse employment settings. Hybrid work patterns may mitigate the impact of chronic pain on work and employee wellbeing.

Key learning points **What is already known about this subject:** 

- The proportion of people living with chronic pain is increasing, leading to significant health, societal and economic burden.
- Supporting people with chronic pain to remain in work is essential to reducing the disability-employment-gap and disability-pay-gap.
- Employers can support people with chronic pain at work, but a lack of awareness of the impact of chronic pain on work and wellbeing can be a barrier to timely action.

**What this study adds:** 

- Chronic pain is associated with work productivity loss, work presenteeism, lower work ability, activity impairments and psychological ill-health.
- Greater pain severity is strongly related to poorer work-related and psychological outcomes.

**What impact this may have on practice or policy:** 

- Employers should raise awareness within their organizations of the impact of chronic pain (across diverse conditions and employment settings) on employees’ work and wellbeing.
- Hybrid working could mitigate the impacts of chronic pain on work and wellbeing, since it is associated with lower pain severity, lower presenteeism and lower work productivity loss.

## Introduction

Chronic pain, defined as pain persisting for more than 3 months, is considered a major public health challenge with a substantial economic and social burden. It affects between one-third and one-half of the population of the United Kingdom (UK), corresponding to just under 28 million adults [1]. Chronic pain is thought to impose costs equivalent to around 4% of a nation’s Gross Domestic Product (GDP) [2]. This impact is amplified as one in four adults in the UK live with multiple health conditions [3]. Chronic pain is rising in the UK working‑age population largely due to increasing long‑term health conditions, especially musculoskeletal (MSK) and mental‑health problems, alongside the ageing demographic and greater levels of work‑limiting illness. UK consensus figures show that chronic pain affects up to 30% of young adults of working age [4], and parliamentary data confirm that 75% of people living with chronic pain are aged 18–65 [5], indicating that the majority are within the working population. Given this substantial working‑age burden—and the explicit focus of UK government, policy groups and major health organizations on addressing the employment impact of chronic pain [6]—the relevance of studying chronic pain in the working population is well‑established and aligned with national priorities. Forty percent of people with chronic pain report being unemployed, a much higher rate than the general unemployment rate in the UK [7]. The Health Foundation’s ‘Commission for Healthier Working Lives’ report highlights that employment plays a key role in protecting and improving people’s health and wellbeing [8]. Supporting people to remain in the active workforce could contribute to reducing the disability-employment gap and disability-pay gap.

The quality and longevity of working life for people with chronic pain can be enhanced with support from employers, such as flexible working arrangements and work adjustments [9]. However, in a survey of employers in England, 46% highlighted ‘a lack of awareness about chronic pain and its impact on work’ as a key barrier to implementing supportive measures [10]. Further, the direct relationship between chronic pain and work participation measures has not been adequately explored. Smith *et al*. have explored the relationship between pain, absenteeism, presenteeism (attending work when unwell) and work productivity loss [11]. However, their study only included individuals with work-related MSK lower body pain and did not include those with pain elsewhere or other causes of chronic pain. Data were collected in 2018, and there have been many changes in the world of work in recent years, including changes to work patterns during and following the coronavirus (COVID-19) pandemic [12] and a dramatic increase in the proportion of people living with chronic pain, currently projected to increase by 32% by 2040 [13]. Research is therefore needed in a contemporary context.

We aimed to examine the relationship between chronic pain and measures of work participation (work ability, work activity and impairment, work self-efficacy, sickness absence, presenteeism and work productivity loss), psychological wellbeing (symptoms of depression and anxiety) and work-related social support. To achieve this, our research questions (RQs) were: RQ1: What are the characteristics of working adults with chronic pain in terms of work participation, psychological wellbeing and work-related social support? RQ2: Do these characteristics differ with pain severity?

## Methods

We analysed cross-sectional baseline data collected as part of a cluster randomized trial in England involving employees living with chronic pain [14]. The University of Nottingham Faculty of Medicine and Health Sciences Research Ethics Committee granted ethics approval on March 31, 2023 (Ref: FMHS 237-0323) and an expanded protocol obtained Health Research Authority approval on July 04, 2024 (IRAS 336655). The trial was registered on May 1, 2023 (ClinicalTrials.gov NCT05838677). Data were collected using an online survey platform between June 2023 and November 2024, reflecting the rolling recruitment of participating organizations into the trial.

Participants were recruited from 18 organizations enrolled in the trial. Eligible participants were aged 18 years or older, employees of an enrolled organization (at time of recruitment) and working in England, and self-identified as experiencing chronic pain. They accessed an online participant information sheet, provided informed consent, and completed the survey independently, without needing to inform their employer.

We accessed data from self-report measures of pain severity, and participant-reported outcome measures (PROMs) for work participation measures and psychological wellbeing.

These measures are appropriate because the constructs of interest are inherently subjective and best obtained through self‑report. Pain lacks a definitive objective marker, and work participation depends on individuals’ perceived abilities, limitations and adaptations, which are not externally observable. Psychological wellbeing reflects internal emotional and cognitive states, while perceived social support is shaped by personal interpretation of social interactions. Validated self‑report instruments therefore provide reliable, sensitive and standardized assessment of these experiences, offering ecologically valid data with minimal participant burden. Pain severity was assessed using a 0–10 numerical rating scale in response to the question: ‘On a scale from 0 to 10, where 0 is no pain and 10 is the worst pain you’ve experienced, at this moment, what number represents your overall pain level?’

*Work-related PROMs included:* (i) The Work Productivity and Activity Impairment Questionnaire (WPAI): General Health V2.0 [15]. Assessed absenteeism, presenteeism, work productivity loss and activity impairment during the past 7 days. Higher percentages indicate greater impairment. Scoring followed the Reilly Associates protocol [15]. Presenteeism was calculated as the percentage impairment while working (Q5 ÷ 10 × 100), and overall work productivity loss combined absenteeism (Q2÷(Q2+Q4)×100) with the remaining time at work adjusted by presenteeism [1−(Q2÷(Q2+Q4))]×(Q5 ÷ 10 × 100)[1−(Q2÷(Q2+Q4))]×(Q5 ÷ 10 × 100)[1−(Q2÷(Q2+Q4))]×(Q5 ÷ 10 × 100), yielding a total percentage of work impaired. (ii) The Work Ability Index (WAI) Item 1 [16]: A single-item numerical rating scale (0–10) asking, ‘Compared to your lifetime best, how would you rate your current work ability?’. Higher score indicates better work ability. (iii) The Work Limitations Questionnaire (WLQ) [17]: Measured presenteeism through 25 Likert-scale items across four domains—time management, physical demands, mental demands and output demands. Scores were converted to percentages (0–100), with higher values indicating greater limitations. Responses marked ‘Does not apply to my job’ were treated as missing and were not included in the final score calculation. (iv) The Work Self-Efficacy Scale (WSE) [18]: Assessed perceived ability to manage chronic pain at work using a 0–10 numerical rating scale. Higher scores indicate higher work self-efficacy. (v) The Demand Control Support Questionnaire Social Support Subscale (DCSQ) [19]: Measured perceived social support. The scale is scored by summing the responses to the six items in the subscale, with each item rated on a 4-point Likert frequency scale. Higher scores on the social support subscale indicate higher levels of perceived social support at work. (vi) Global items [14, 20]: Included single-item measures of job satisfaction and job stressfulness (Likert scale), and turnover intentions (yes/no). Job satisfaction was dichotomized into high satisfaction (satisfied/extremely satisfied) and low satisfaction (extremely dissatisfied/dissatisfied/neither). Job stressfulness was dichotomized into high stress (very/extremely stressful) and low stress (not at all/mildly/moderately). (vii) Sickness absence: Measured using a Likert-scale response to the question, ‘During the past 12 months, how many days have you had off work in total because of problems with your health?’ Options included: ‘no time’, ‘less than 5 days’, ‘5–20 days’, ‘more than 20 days’ or ‘not applicable’ (not working during this time). This was dichotomized to (none/any) sick leave. (viii) Work Patterns: Likert scale responses gathered information about the frequency of work patterns of individuals. Responses were ‘always’, ’very often’, ’sometimes’ or ’never’ in response to whether respondents do the following: overtime, flexitime, flexible hours, fixed hours, shifts, travel (away from home), driving (for work), home-based and hybrid working. These were scored as ‘always’ and ‘very often’ equals 1, and other responses as 0.

*Psychological PROMs included:* (i) Patient Health Questionnaire (PHQ-2) [21]: Assessed symptoms of depression. The measure is scored from 0 (not at all) to 3 (nearly every day), resulting in a total score ranging from 0 to 6. A score of 3 was used as a conservative screening threshold for depression based on a study in which employees reported a mean (SD) of 1.62 (1.67) [22]. (ii) General Anxiety Disorder Scale (GAD-7) [23]: Assessed the severity of anxiety. The score is calculated by assigning scores of 0, 1, 2 and 3 to the response categories of 'not at all’, 'several days’, 'more than half the days’ and 'nearly every day’, adding up to a possible total of 21. Scores of 5, 10 and 15 were taken as cut-off points for mild, moderate and severe anxiety, respectively. A study with employees reported a mean (SD) of 5.42 (5.24) [22].

Demographic data and covariates (age (in years), gender and body mass index (BMI) classification) were included in all multivariable analyses. BMI was calculated from self-reported weight (kg) and height (m) and categorized using World Health Organization (WHO) classifications [24]. Obese subcategories were collapsed into a single category of (BMI ≥30).

Descriptive statistics included means with standard deviations, or medians and ranges for non-normally distributed variables, and frequencies and percentages for categorical variables. Normality was assessed using histograms and the Shapiro–Wilk test. Group differences were analysed using the Wilcoxon rank-sum test or *t*-test, as appropriate. Cases with missing data were excluded from regression analyses. Multivariable linear regression models were used to examine associations between pain severity and work participation measures, psychological wellbeing (PHQ-2 and GAD-7), work-related social support (DCSQ), age, gender and BMI classification. Model assumptions were assessed before analysis. Analyses were conducted using STATA vSE18.5 [25], with a significance level set at 0.05.

## Results

Following eligibility screening, 12 individuals were excluded, resulting in 380 eligible participants who received a survey link. Of these, 310 employees (82%) completed the survey (82% female, 15 ethnicities represented, 91% White) (Figure 1). Average age was 46.3 years (SD = 11.2), range 21 to 69 years. The majority identified as female (*n* = 254; 82%). Demographic and employment characteristics are summarized in Table 1.

**Figure 1:**
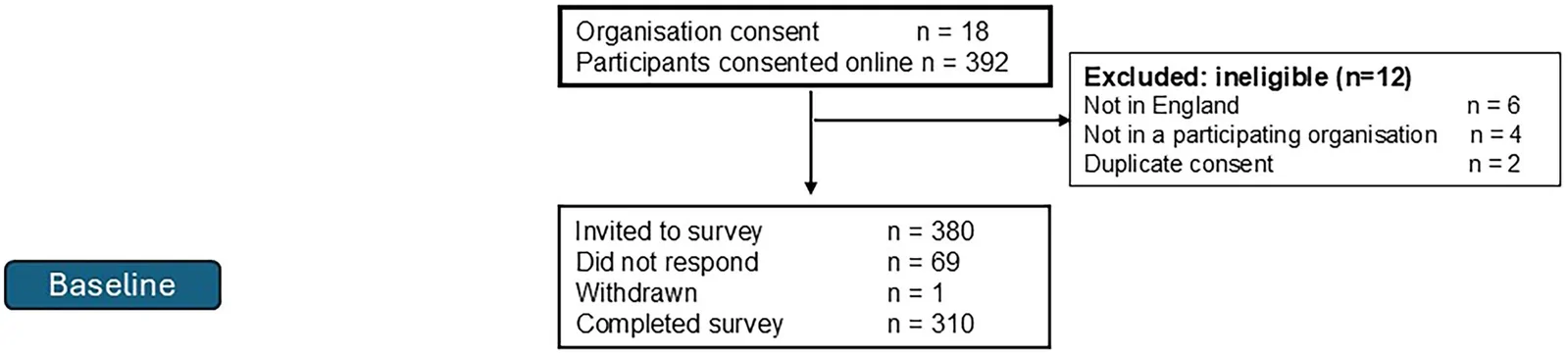
CONSORT flow diagram from the Pain-at-Work trial.

**Table 1:** Demographic and employment characteristics

Characteristic	Total (*n* = 310)	Comparator: England employees
Age (years): mean [SD]	46 [11]	42%
Gender: *n* (%)		
Female	254 (82%)	49%
Male	51 (16%)	51%
Non-binary	5 (2%)	
Living arrangements: *n* (%)		
Married/living with partner	262 (84%)	
Living alone	43 (14%)	
Other living arrangements	5 (2%)	
Ethnicity: *n* (%)		
Black-Caribbean	2 (0.6%)	0.8%
Chinese	3 (1.0%)	NA
Filipino	2 (0.6%)	NA
Indian	8 (2.6%)	1.1%
Mixed race	5 (1.6%)	1.2%
Other	5 (1.6%)	0.7%
Pakastani	2 (0.6%)	0.8%
White	283 (91%)	50%
Body mass index (kg/m^2^)^a^		
Under-weight <18.5	1 (0.3%)	
Healthy weight 18–24.9	71 (23.7%)	
Over-weight 25–29.9	87 (29%)	31%
Obese >30	143 (47%)	29%
Education: *n* (%) highest reported		
No formal qualification	2 (1%)	
O Level/GCSE or equivalent	19 (6%)	
A Level or equivalent	14 (4%)	
Vocational training certificate(s)	77 (25%)	
University degree or HND	130 (42%)	
Higher professional qualifications	68 (22%)	
School leaving age (years): median [IQR]	16.5 [15–26]	
Direct from school to full-time education or university: *n* (%)	106 (34%)	
Income bands: *n* (%)		
£0–£5,199 per year	3 (1%)	
£5200–£7799 per year	0 (0%)	
£7800–£12 999 per year	5 (2%)	
£13 000–£18 199 per year	7 (2%)	
£18 200–£23 399 per year	22 (7%)	
£23 400–£31 199 per year	44 (14%)	Average £35 000
£31 200–£38 999 per year	41 (13%)	
£39 000 or more per year	187 (61%)	
Years in current main job: mean [SD]	4.4 [0.8–39.3]	
Full-time work (>35 h/week) *n* (%)	201 (68%)	
Average hours worked/week: mean [SD]	33.9 [6.4]	
Pain duration (years): median [IQR]	5.8 [0.8–40.8]	

Other ethnicities include one each for: Mauritian, Mexican Indigenous, Latino British, Mixed White Gypsy, West Indian.

Comparator data sources: age [26], gender [27], ethnicity [28], BMI [29], and average earnings [30].

IQR, interquartile range; Ref, reference; SD, standard deviation.

a BMI was classified according to the WHO guidelines [20].

All but one participant reported experiencing pain at the time of survey completion. Mean pain severity score was 5.2 (SD = 1.9), considered to be ‘moderate’ [31]. Duration of chronic pain ranged from a few months to 51 years, with a median of 5.83 years. Participants reported 383 health conditions potentially contributing to their chronic pain, including MSK, migraine, gynaecological and autoimmune disorders.

Participant-reported outcomes regarding work-related characteristics are reported in Table 2 (available as Supplementary data at *Occupational Medicine* Online). Respondents to the WPAI reported the mean number of hours worked for the previous 7 days, which was 31.8 (SD = 12.9), fewer than the national average 36.5 h/week [32]. On average, 2.2 h/week were reported lost due to health-related issues. Their self-reported work productivity loss was 46.4% (SD = 27.0), whilst activity impairment was 51.1% (SD = 26.0). Average health-related absenteeism was 7.6%, while presenteeism averaged 44.3% (SD = 26.3). All outcomes, except absenteeism, were significantly and positively associated with pain severity—higher pain severity was linked to greater impairment and presenteeism (Table 2 (available as Supplementary data at *Occupational Medicine* Online), Figure 2).

**Figure 2:**
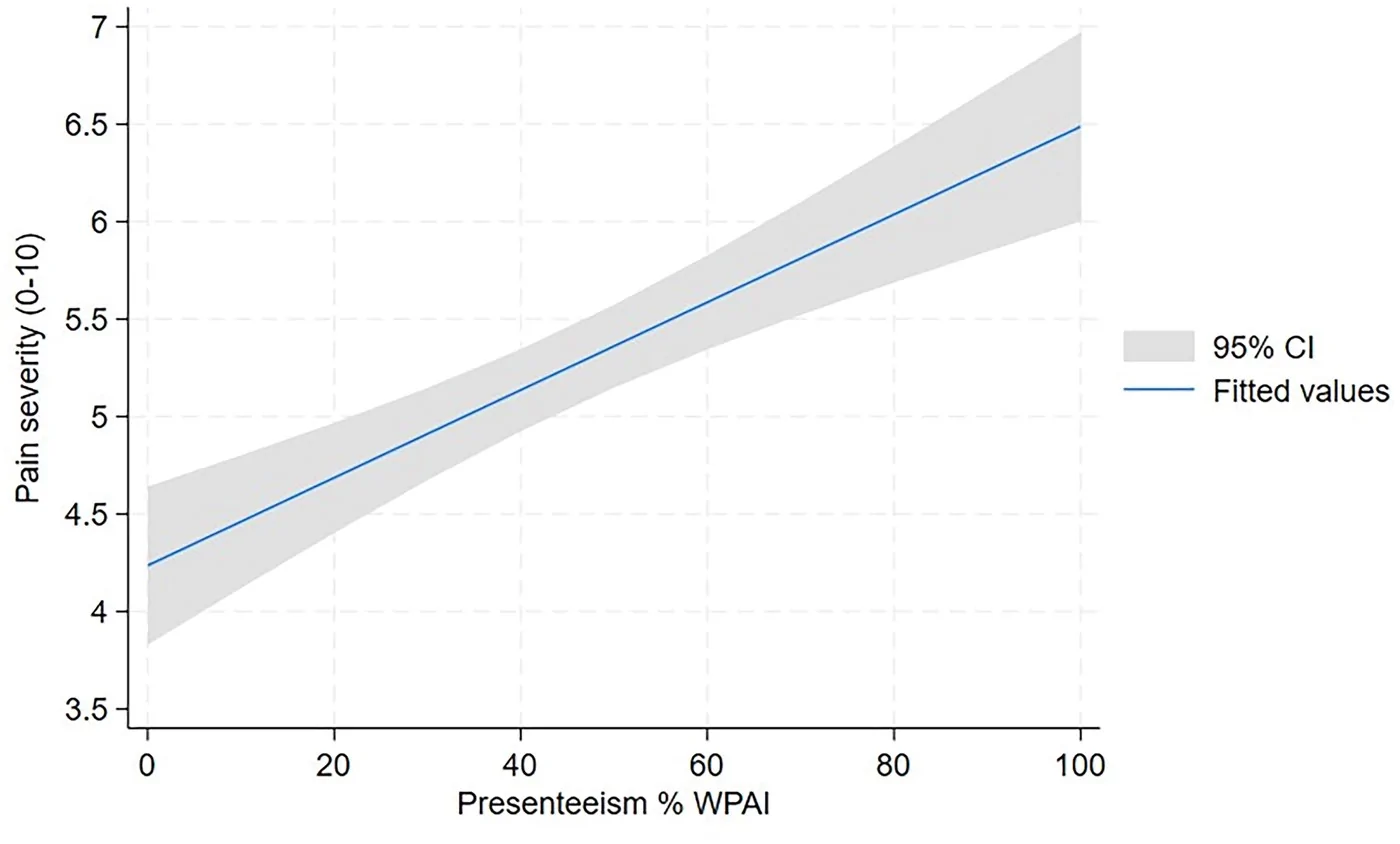
The association of pain severity with presenteeism.

Using the WAI, participants rated their current work ability compared to their lifetime best at a mean of 6.4 (SD = 1.9) on a 0–10 scale, considered ‘moderate’ [33]. This score was significantly negatively associated with pain severity; individuals with higher pain severity reported a lower lifetime best score compared to those with lower pain severity (Table 2, available as Supplementary data at *Occupational Medicine* Online).

Participants reported similar levels of limitation across all four WLQ domains. However, only the physical demands scale (mean = 43.6, SD = 21.9) showed a statistically significant positive association with pain severity—greater pain severity was associated with greater limitations (see Table 2, available as Supplementary data at *Occupational Medicine* Online). There was no significant relationship between pain severity and perceived social support at work.

Of the respondents, 174 (58%) reported ‘high satisfaction’ with their job. Furthermore, 58 (19%) described their job as ‘high stress’. Job satisfaction, job stressfulness, turnover intention and categories of days off sick were not significantly associated with pain severity (Table 2, available as Supplementary data at *Occupational Medicine* Online).

Of PHQ-2 completers, 108/288 (38%) scored 3 or more, indicating ‘possible major depressive disorder’ [21]. The average GAD-7 score was 7.93 (SD 5.69), classified as ‘mild anxiety’ [23]. Higher PHQ-2 and GAD-7 scores were significantly associated with higher pain severity (Table 2, available as Supplementary data at *Occupational Medicine* Online).

There was no significant relationship between pain severity and working nights, shifts, fixed hours, flexitime, overtime, work-related travel, home-based work or driving for work (Table 2, available as Supplementary data at *Occupational Medicine* Online). Hybrid working (working flexibly between work premises and home) was experienced ‘always’ or ‘very often’ by 140 (47%) participants and was negatively associated with pain severity (*β* = −0.79, 95% CI −1.22 to −0.37), *P* < 0.001 (Table 2, available as Supplementary data at *Occupational Medicine* Online). Hybrid working was associated with a significantly lower score for presenteeism (measured using the WPAI) (*β* =−7.61, 95% CI –13.57 to −1.63), *P* = 0.013, and lower work productivity loss (WPAI) (*β* =−6.72, 95% CI −12.94 to −0.49), *P* = 0.035. WPAI sickness absenteeism and activity impairment, WAI and WLQ were not significantly associated with hybrid working.

Regarding sociodemographic factors, older school leaving age (*β* = −0.15, 95% CI −0.27 to −0.04), *P* = 0.009 and level of highest qualification (*β* =−0.32, 95% CI −0.52 to −0.13), *P* = 0.001 were both significantly and negatively associated with pain severity. Other sociodemographic factors including age, gender, living arrangements, income band, employer, occupation type and contract type were not statistically associated with pain severity.

In multivariable regression analyses adjusted for psychological wellbeing (PHQ-2 and GAD-7), work-related social support (DCSQ), hybrid working, age, gender and BMI classification, higher pain severity was significantly associated with several adverse work-related outcomes. Specifically, higher pain severity was associated with increased presenteeism (*β* = 3.67, 95% CI 2.08–5.27, *P *< 0.001); higher work productivity loss (*β* = 4.79, 95% CI 2.13–7.44, *P = *0.001) and greater activity impairment (*β* = 4.93, 95% CI 3.41–6.44, *P *< 0.001). Within the WLQ, higher pain severity was significantly associated with increased limitations in the physical demand domain (*β* = 3.92, 95% CI 2.64–5.21, *P *< 0.001). For full models, see Table 3. When psychological wellbeing (depression/anxiety) and social support were included in the model, pain severity was not statistically associated with work ability, as measured by the WAI. Multicollinearity was evaluated using variance inflation factors (VIF), with all values below 2.2, indicating no concern. Residuals were tested for normality using skewness and kurtosis tests, confirming normal distribution for each model.

**Table 3:** Association of pain severity with presenteeism, work productivity loss, activity impairment and the physical demand scale

		**Outcome variable (beta coefficient (95% CI))**			
Variable	Interval/category	Presenteeism (WPAI) (*n* = 281)	Work productivity loss (WPAI) (*n* = 276)	Activity impairment (WPAI) (*n* = 284)	Physical demand (WLQ) (*n* = 282)
Pain severity	0–10	3.67 (2.08, 4.54), *P* < 0.001	4.79 (2.13, 7.44), *P* = 0.001	4.93 (3.41, 6.44), *P* < 0.001	3.92 (2.64, 5.21), *P* < 0.001
PHQ-2	0–6	1.29 (−0.88, 3.46), ns	1.83 (−1.55, 5.21), ns	2.25 (0.16, 4.34), *P* = 0.035	2.85 (1.10, 4.60), *P* = 0.001
GAD-7	0–21	0.30 (−0.42, 1.01), ns	−0.04 (−1.15, 1.07), ns	0.33 (−0.36, 1.02), ns	0.28 (−0.29, 0.86), ns
DCSQ social	6–24	−0.94 (−1.91, 0.02), ns	−1.76 (−3.28, −0.25), *P* = 0.023	−0.42 (−1.34, 0.50), ns	−0.11 (−0.89, 0.66), ns
Hybrid working	No/yes	−3.82 (−9.66, 2.02), ns	−3.47 (−12.43, 5.50), ns	6.58 (0.99, 12.16) *P* = 0.021	−1.04 (−5.72, 3.65), ns
Gender	Female	Ref	Ref	Ref	Ref
	Male	2.22 (−5.35, 9.79), ns	4.32 (−16.80, 8.15), ns	−1.08 (−8.34, 6.18), ns	−3.07 (−9.16, 3.01), ns
	Non-binary	5.06 (−15.62, 27.97), ns	−36.23 (−97.27, 18.81), ns	17.28 (−3.70, 38.26), ns	−4.24 (−21.82, 13.33), ns
Age (years)	Years	−0.28 (−0.55, −0.01), *P* = 0.039	−0.05 (−0.46, 0.36), ns	0.02 (−0.24, 0.27), ns	0.11 (−0.11, 0.33), ns
BMI class^a^					
	Healthy	Ref	Ref	Ref	Ref
	Underweight	−25.92 (−73.65, 21.81), ns	−52.11 (−106.62, 2.40), ns	−0.68 (−46.55, 45.18), ns	24.53 (−13.86, 62.92), ns
	Overweight	−0.20 (−8.04, 7.63), ns	0.84 (−10.84, 12.51), ns	3.53 (−3.97, 11.03), ns	1.26 (−5.02, 7.54), ns
	Obese	2.13 (−5.06, 9.33), ns	11.94 (1.36, 22.52), *P* = 0.027	6.53 (−0.35, 13.40), ns	2.41 (−3.36, 8.18), ns
*r* ^2^		0.1820	0.2330	0.2500	0.2652

Data are the beta coefficients (95% CI). Each model is the outcome variable (percentage) adjusted for pain, mood (PHQ-2, GAD-7), social support (DCSQ), hybrid working, age, sex and BMI class. The outcome variables presenteeism, work productivity loss and activity impairment were reported in the Work Productivity and Activity Impairment Questionnaire: General Health V2.0 (WPAI: GH). Physical demand scale was reported in the Work Limitations Questionnaire (WLQ).

BMI, body mass index; 95% CI, confidence intervals; DCSQ, Demand Control Social Support Questionnaire social support subscale; GAD-7, General Anxiety Disorder scale; PHQ-2, Patient Health Questionnaire; Ref, reference group.

a WHO classification for BMI (kg/m^2^), underweight, <18.5; normal, 18.5–24.9; pre-obese, 25–29.9 and obese, >30.

## Discussion

This is a novel study examining the relationship between chronic pain and work participation in a sample of UK employees with diverse chronic pain conditions from different employment settings. We found that working-age adults living with diverse chronic pain conditions experienced significant work productivity loss (46.4%, average 2.2 h/week), work presenteeism (44.3%) and lower work ability, alongside activity impairment and impacts on physical work demands. Overall, this means that a high proportion of participants in this survey attended work while unwell. They worked fewer hours than the national average and lost about 2 h of work each week because of their health. They also reported that their pain reduced their productivity by nearly half and made everyday activities harder to carry out. Psychological ill-health was prevalent, with most employees in our sample reporting at least mild anxiety, and over one third reporting symptoms of depression.

Although pain levels varied, on average, participants experienced pain they reported to be ‘moderate’. Importantly, greater pain severity was adversely associated with poorer work outcomes irrespective of psychological wellbeing. Hybrid working appeared to be protective, as it was associated with lower pain severity, lower presenteeism and lower work productivity loss; other work patterns (overtime, flexitime, flexible hours, fixed hours, shifts, travel away from home, driving for work, home-based) were unrelated to pain severity or work participation in our sample.

It would be useful to compare our findings with those of a ‘typical worker’; however, no directly comparable UK data currently quantify work productivity loss using a validated measure such as the WPAI in the general working population. Although UK‑involved WPAI studies exist, these are limited to patient groups (e.g. the EULAR‑PRO Worker Productivity Study), while UK workplace productivity studies do not use WPAI or comparable survey‑based productivity measures.

Our findings need to be considered alongside some limitations. Since data were drawn from baseline surveys in a clinical trial, rolling recruitment meant that participants completed the surveys at different times throughout the year. Since this is cross-sectional data, we can only determine associations, not causality. More women participated than other gender identities; this likely reflects the higher prevalence of chronic pain in women [34], and/or could relate to gender differences in pain reporting, health-related stigma, or research participation. Almost half the sample self-reported obesity, which is unsurprising given that 64.5% of adults in England are overweight or obese [35] and obesity is a significant and well‑established risk factor for chronic pain [36]. Given the sample size and the 383 conditions represented, participant numbers per condition were insufficient for meaningful comparisons between specific pain conditions and workplace management strategies. While this highlights the diversity of chronic pain, future studies with larger samples could undertake subgroup analyses. Although 15 ethnicities were represented, the greatest proportion of the sample was White and 61% reported above average income. However, neither ethnicity nor income level was associated with pain severity, work-related or psychological measures. Participants were drawn from a trial which enrolled predominantly large employers–our sample therefore includes few individuals from smaller organizations which is a common challenge in organizational and occupational research.

All measures and exposures are self-reported, by the nature of the research. It cannot be excluded that an individual’s pain impacts how they respond to self-report measures. We used multiple PROMs to capture the multidimensional nature of pain, work participation and psychological wellbeing. These constructs cannot be adequately represented by a single measure, and each PROM was selected for its distinct contribution to the conceptual framework of the study.

The economic burden of chronic pain has been demonstrated in prior research conducted in the United States [37], which identified work productivity loss with chronic pain, that was mostly (76.6%) explained by reduced performance while at work rather than work absence. In our study, health-related absence was relatively low, but work presenteeism was high, supporting these findings. Health has been described as ‘the most important driver of presenteeism in the UK’ [38], with any kind of physical impairment estimated to lead to a doubling (e.g., from 7% to 14%) in the probability of reduced productivity at work [39]. This has implications for organizations since the cost of presenteeism to business is double that of absenteeism [39].

Our findings suggest that hybrid working could have distinct benefits for both individuals (lower pain) and organizations (lower presenteeism and lower productivity loss). Hybrid working has become more commonplace post-pandemic, although research on hybrid working often focuses on practical benefits for employees and/or organizational culture without consideration of productivity and performance [40]. Our study provides indicative evidence to suggest that hybrid working could be a protective factor with regards the impact of chronic pain on presenteeism and productivity. Where feasible, employers could offer hybrid working arrangements to support employees with chronic pain. This is supported by findings from studies with broader employee samples [41] and specifically, workers with chronic pain [42], which show that employees perceive hybrid work arrangements as a valuable job resource or job demand enabler to support health and wellbeing. Employers implementing hybrid work patterns must seek to ensure it is managed inclusively, to avoid exacerbating existing inequalities through risk of stigma, isolation and unequal access to resources [43]. If hybrid working is not possible for some employees, employers can explore alternative strategies (e.g., fully remote or flexible work arrangements) where feasible, tailored to workers’ needs; albeit such work arrangements are more feasible in some work/industry types than others.

In conclusion, chronic pain significantly impacts work presenteeism and productivity. With its growing prevalence across the population, this creates a compelling business case for employers to adopt policies and practices that support employees to manage chronic pain effectively at work. Our findings show that hybrid working may help mitigate its impact on performance and productivity. However, this form of job design is not feasible in all roles, and many employees will require tailored support to participate fully and effectively in the workplace.

## Supplementary Material

kqag079_Supplementary_Data

## Data Availability

The data that support the findings of this study are available from the corresponding author upon reasonable request.
