# Supplementary material for: Chronic pain and its relationship with work participation
Source: Occup Med (Lond). 2026 Sep 7;76(7):470–8. doi: 10.1093/occmed/kqag079 (PMC13740503; doi:10.1093/occmed/kqag079)
Supplement: Supplementary file 1 [file kqag079_supplementary_data.pdf]

Table 2 Participant-reported outcomes and bivariate associations with pain severity

Outcome	Total (n = 310)	Beta coeff (SE)	P value
Pain severity: mean [SD] (0–10)	5.22 [1.92], (n = 307)	NA	NA
Work Productivity and Activity Impairment Questionnaire: General Health V2.0 (WPAI: GH): mean [SD] %	(n = 295)		
Absenteeism	7.60 [20.23]	0.92 (0.63)	ns
Presenteeism	44.34 [26.28]	4.36 (0.77)	<0.001
Productivity loss	46.44 [27.02]	4.38 (0.81)	<0.001
Activity impairment	51.01 [26.23]	5.25 (0.74)	<0.001
Work Ability Index (WAI): mean [SD]	(n = 298)		
Compared with your lifetime best, how would you rate your current work ability? (0–10)	6.37 [1.86]	−0.12 (0.06)	0.038
Workplace Limitations Questionnaire (WLQ): mean [SD]	(n = 288)		
Time management demands	46.67 [24.99]	1.25 (0.78)	ns
Physical demands	44.64 [21.89]	4.87 (0.63)	<0.001
Mental/interpersonal demands	43.49 [26.53]	0.22 (0.83)	ns
Output demands	43.87 [29.15]	0.92 (0.91)	ns
Work Self-Efficacy (WSE): mean [SD] (0–10)			
Currently	(n = 298)		
I feel confident that I can work	7.28 [2.49]	−0.17 (0.08)	0.022
I feel confident in my ability to manage my condition in a work environment	6.38 [2.41]	−0.14 (0.07)	ns
I feel confident that, in general, working would not make my condition worse	5.21 [2.87]	−0.03 (0.09)	ns
In the next year			
How motivated/interested are you to continue to work?	7.92 [2.64]	−0.12 (0.08)	ns
How important is it to you to continue to work?	8.99 [2.02]	−0.07 (0.06)	ns
How confident are you that you can continue to work?	7.44 [2.40]	−0.17 (0.07)	0.017
Demand Control Support Questionnaire (Social Support at Work subscale) (DCSQ)	(n = 297)		
Mean [SD]	20.72 [3.10]	0.015 (0.53)	ns
Job satisfaction: n (%)[a]	(n = 298)		
Low satisfaction	124 (42%)		
High satisfaction	174 (58%)	−0.06 (0.06)	ns
Job stressfulness: n (%)[a]	(n = 298)		
Low stress	240 (81%)		
High stress	58 (19%)	−0.12 (0.08)	ns
Turnover intention: n (%) yes, considering leaving job[a]	88 (30%), (n = 298)	0.09 (0.07)	ns
Sickness absence in the past 12 months: n (%)	(n = 298)		ns
None	58 (19%)		
Any sick leave	240 (81%)	0.16 (0.08)	ns
Patient Health Questionnaire (PHQ-2) score ≥3: n (%)	108 (38%)		
Mean [SD]	2.28 [1.81]	0.22 (0.06)	<0.001
General Anxiety Disorder (GAD-7): mean [SD] (0–21)	7.93 [5.69] n = 288	0.62 (0.17)	<0.001
Minimal anxiety (0–4)	97 (33%)		
Mild anxiety (5–9)	88 (31%)		
Moderate anxiety (10–14)	58 (20%)		
Severe anxiety (≥15)	45 (16%)		
Further work patterns: always or often: n (%)	(n = 299)		
Overtime	68 (23%)	−0.34 (0.47)	ns
Flexitime	86 (29%)	−0.20 (0.25)	ns
Fixed hours	157 (53%)	−0.03 (0.22)	ns
Shift work	37 (12%)	0.36 (0.34)	ns
Night work	13 (4%)	−0.21 (0.54)	ns
Travel (away from home)	16 (5%)	0.18 (0.49)	ns
Driving (for work)	77 (26)	−0.43 (0.25)	ns
Home-based worker	77 (26%)	0.04 (0.25)	ns
Hybrid working	140 (47%)	−0.79 (0.22)	<0.001

For all variables, higher scores indicate worse work/health status, apart from work self-efficacy, where higher scores indicate increased confidence/motivation. Non-significant trend (P = 0.066). The number of observations varied across variables; however, 288 people completed all variables.
Beta coeff, Beta coefficient; NA, not applicable; ns, non-significant; SD, standard deviation; SE, standard error.
[a]Categories were collapsed into dichotomous variables, and logistic regression was performed instead of linear regression.
